# Effects of *Rhodotorula mucilaginosa* on the Immune Function and Gut Microbiota of Mice

**DOI:** 10.3389/ffunb.2021.705696

**Published:** 2021-08-18

**Authors:** Ye Ge, Kaisen Huang, Weitian Xie, Chunhou Xu, Qiucheng Yao, Ying Liu

**Affiliations:** ^1^College of Coastal Agricultural Sciences, Guangdong Ocean University, Zhanjiang, China; ^2^College of Food Science and Technology, Guangdong Ocean University, Zhanjiang, China

**Keywords:** *Rhodotorula mucilaginosa*, immune function, intestinal flora, marine yeasts, microbial fermentation

## Abstract

As a dominant species among marine yeasts, *Rhodotorula benthica* accounts for ~50% of all marine yeasts. *Rhodotorula* is rich in a variety of bioactive substances and commonly used in the production of carotenoids by microbial fermentation and is worth developing. Therefore, the present study used a strain of *Rhodotorula mucilaginosa* isolated from the coastal waters of the South China Sea as the target yeast to investigate its impact on the immune function and gut microbiota of mice. A total of 200 mice were randomly divided into gavage groups and control group and garaged for 30 consecutive days at different concentration. Samples were collected on day 15 and day 30 of gavage administration. The results showed that *R. mucilaginosa* ZTHY2 could increase the thymus and spleen indices of mice, and its effect on the thymus index was more significant after long-term gavage administration. Short-term (15 days) gavage administration of *R. mucilaginosa* suspension enhanced delayed hypersensitivity in mice, increased serum IgG, IgA, and IL-2. Long-term (30 days) gavage administration of *R. mucilaginosa* suspension significantly enhanced the phagocytosis of macrophages in mice and significantly increased serum TNF-α and INF-γ. *R. mucilaginosa* ZTHY2 altered the structure of the gut microbiota of mice at the phylum and genus levels, leading to an increased relative abundance of Firmicutes and *Lactobacillus* and a decreased relative abundance of Bacteroidetes. This strain increased the beneficial intestinal bacteria and reduced the harmful intestinal bacteria in mice. This study provides experimental evidence and lays the foundation for the future development and application of this strain as a microecological source of carotenoids.

## Introduction

*Rhodotorula* is a genus of eukaryotic microorganisms in the family Sporidiobolaceae (order *Sporidiales*, class *Pucciniomycetes*, phylum *Basidiomycota*) that is widely found in animals, plants, rivers, lakes, and oceans (Falces-Romero et al., 2016; Cudowski and Pietryczuk, 2019; Daudu et al., 2020). Cells of *Rhodotorula* not only are rich in conventional nutrients such as carotenoids, proteins, and polysaccharides but also contain amino acids, polyunsaturated fatty acids, vitamin E, nucleotides, and astaxanthin (Gupta et al., 2012). *Rhodotorula mucilaginosa* is a dominant species in coastal waters of the South China Sea (Hagler and Mendonca-Hagler, 1981; Laurie et al., 2009).

As a good feed additive, *Rhodotorula* can improve animal performance. However, the research and application of *Rhodotorula* as a microecological agent are mainly focused on aquatic animals, and few reports on livestock and poultry (Lario et al., 2015). *Rhodotorula*, especially originated Marine had sources (Hagler and Mendonca-Hagler, 1981), had many good applications, such as easy separation and cultivate, grow fast, rich metabolic product. It is a good live strain, which had the effect on the animal body nutrition effect, immune function, antioxidant function, which has a broad market prospect and greater economic benefits as preparation of probiotics (Guo et al., 2020).

Yeast is not a strain of the normal microbiota in the intestinal tract of animals, but a kind of passing bacteria in the gastrointestinal tract (Yang et al., 2014). It can maintain metabolic activity through the acidic environment in the stomach smoothly, and consume oxygen in the intestinal tract through respiration. The beneficial bacteria in the intestinal tract are anaerobic bacteria, while most of the pathogenic bacteria are aerobic bacteria. Yeast inhibits the growth and reproduction of pathogenic bacteria by consuming the residual oxygen in the intestinal tract through respiration, to promote the growth of beneficial bacteria (Shi-Ping et al., 2010).

Yeast in the gut increases the repertoire of microorganisms interacting with the host intestine, which could influence health and disease (Raggi et al., 2014). There was a report that *Rhodotorula mucilaginosa* solid-state fermentation product supplementation improved the laying performance, egg quality and intestinal microflora of hens (Sun et al., 2020). However, there are few reports on the specific mechanism of red yeast action on animal intestinal flora, and further studies are needed.

This study investigated a probiotic strain, *Rhodotorula mucilaginosa* ZTHY2, isolated and screened from coastal waters of the South China Sea, to evaluate its effect on the immune function and gut microbiota of mice based on increasing carotenoid production, to provide empirical evidence for this strain as a microecological source of carotenoids.

## Experiment Methods

### Strain

*R. mucilaginosa* ZTHY2 was isolated, identified, screened, and preserved by the Preventive Veterinary Medicine Laboratory of Guangdong Ocean University (Sun et al., 2015).

### Effect of *Rhodotorula mucilaginosa* on the Immune Function of Mice

Kun Ming mice were randomly classified (gavage groups and control group), each with 20 female mice and 20 male mice *R. mucilaginosa* suspensions at concentrations of 2 × 10^6^ CFU/mL, 2 × 10^7^ CFU/mL, 2 × 10^8^ CFU/mL, and 2 × 10^9^ CFU/mL were administered by gavage in the gavage groups, respectively (0.5 mL/time, once a day, for 30 consecutive days). Sterilized saline was administered in control group. On days 15 (named A group) and 30 (named B group), three mice were selected for measurement of each parameter. The spleen, thymus, liver, and kidney of mice were weighed and isolated, and the organ indices were calculated. Delayed-type hypersensitivity (DTH) was measured by dinitrofluorobenzene smear and ear weighing method. The phagocytic function of macrophages of the mice was determined by carbon clearance assay. The concentrations of serum immunoglobulin G (IgG), immunoglobulin A (IgA), interleukin (IL)-2, tumor necrosis factor-α (TNF-α), interferon-γ (IFN-γ), cluster of differentiation (CD) 4, and CD8 in mice were determined by ELISA kits (Nanjing Jiancheng Bioengineering Institute) according to the instructions. The regression equation of the standard curve was calculated from concentration and optical density, and the contents were also calculated.

### Effect of *Rhodotorula mucilaginosa* on the Gut Microbiota of Mice

R. mucilaginosa suspensions at the above concentrations were administered to the mice by gavage, and the mice were classified into five groups (same as above). On days 15 (control group A1 and gavage groups A2, A3, A4, and A5) and 30 (control B1 group and gavage groups B2, B3, B4, and B5), the intestinal contents of mice were collected and sent to the Sangon Biotech (Shanghai). 16S rDNA gene sequencing and analysis were used to detect the total DNA of the gut microbiota (Oh et al., 2016; Li et al., 2020). Mothur software was used to cluster the operational taxonomic units (OTUs) from the sequences by Usearch. The α diversity was calculated using QIIme (Version 1.9.1) software, including Chao1 index, ACE index, Shannon index and Simpson index. The richness indices of gut microbial community distribution [Chao1 and abundance-based coverage estimator (ACE)] and the diversity index (Shannon index, Simpson index and Coverage) of the community distribution were calculated (Chen et al., 2013). The Chao1 index and Ace index were used to calculate the abundance of bacteria, and the Shannon index and Simpson index were used to calculate the diversity of bacteria. The higher Shannon index value, the higher microbial community diversity. The higher the Simpson index value, the lower the microbial community diversity; The library Coverage of each sample is represented by the Coverage Index, which can reflect whether the sequencing results can represent the actual situation of the sequencing samples. The higher the value, the lower the probability of the sequence not detected in the sample. The GPlots package of R was used to draw a heat map of the abundances of gut microbes of mice. Uchime software was used to identify and knock out chimeras. The RDP classifier was used to annotate the species classification for each sequence. Blastn was compared with Silva database with 97% confidence interval (CI). Species classification of each OTU was carried out to obtain information on the microbial classification at different taxonomic levels. According to the number of sequences in each OTU, the abundance table of OTUs in each sample was generated for subsequent analysis.

### Statistical Analysis

The results are expressed as the mean value ± standard deviation. SPSS 22.0 was used to analyze the significance of differences and to make multiple comparisons (Duncan test) (Fierer et al., 2007). The significance level was set at *P* < 0.05.

## Experimental Results

### Effect of *Rhodotorula mucilaginosa* on Immune Function of Mice

#### Functional Index of the Organs

**Figure 1:**
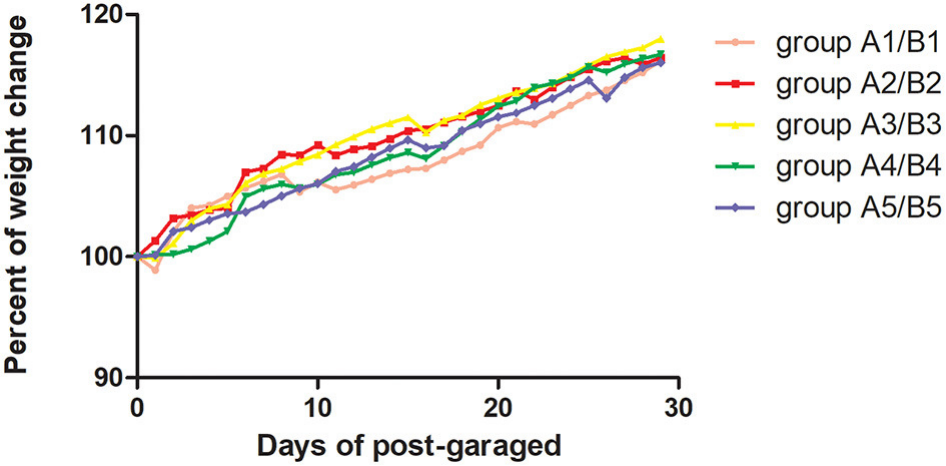
The present of body weight change of mice was tested every day. The body weight was the average of 40 mice in one group. Pink curve was group A1/B1 with Sterilized saline by gavage, red curve was group A2/B2 with 2 × 10^6^ CFU/mL by gavage; Blue curve was group A3/B3 with 2 × 10^7^ CFU/mL by gavage, green curve was group A4/B4 with 2 × 10^8^ CFU/mL by gavage, and deeply purple curve was group A5/B5 with 2 × 10^9^ CFU/mL by gavage.

On day 15 of gavage administration of *R. mucilaginosa* suspension, there was no significant difference in the functional index of the spleen, thymus, liver, or kidney between the four gavage groups and control group, whereas there was a significant difference in the spleen index between group A4 and group A5 (*P* < 0.05). Group A4 had a 26.2% higher spleen index and 12.86% higher thymus index than group A1. On day 30 of gavage administration, there was no significant difference in the spleen index but a significant difference in the thymus index between the four gavage groups and group B1 (*P* < 0.05); the thymus index in group B2 was 37.02% higher than that in group B1; the liver index in groups B2, B3, and B4 and the kidney index in groups B2, B3, and B4 were significantly different from those in group B1 (*P* < 0.05) (Table 1). The body weight of mice was tested every day (Figure 1).

**Table 1:** Effects of *Rhodotorula mociliginosa* on mouses organs (15 days and 30 days data).

**Groups**	**Spleen index**	**Liver index**	**Thymus index**	**Kidney index**
Control group A1	4.4329 ± 0.3962^ab^	51.4665 ± 1.5802^a^	2.5560 ± 0.2804^a^	12.4637 ± 0.8290^a^
Gavage group A2	4.1801 ± 0.5798^ab^	51.3714 ± 3.9373^a^	2.4993 ± 0.2753^a^	11.2444 ± 0.5227^a^
Gavage group A3	4.8437 ± 0.5025^ab^	53.8740 ± 2.6333^a^	2.5017 ± 0.2436^a^	11.7034 ± 0.6398^a^
Gavage group A4	5.5946 ± 0.6700^a^	55.5966 ± 1.4285^a^	2.8846 ± 0.2511^a^	11.7208 ± 0.1548^a^
Gavage group A5	4.3217 ± 0.5747^bc^	49.1879 ± 1.7348^a^	2.4033 ± 0.2245^a^	11.4398 ± 0.3331^a^
Control group B1	5.9858 ± 1.2300^a^	57.9662 ± 1.9191^a^	3.0744 ± 0.5608^a^	12.9843 ± 0.9876^a^
Gavage group B2	4.4189 ± 0.2679^a^	49.7391 ± 1.4902^b^	4.2125 ± 0.2913^b^	11.6905 ± 0.5287^b^
Gavage group B3	4.6957 ± 0.5026^a^	50.2408 ± 0.9541^b^	3.3814 ± 0.2690^c^	12.5676 ± 0.4962^a^
Gavage group B4	4.9717 ± 0.3056^a^	48.4020 ± 1.0566^b^	3.0795 ± 0.2388^c^	11.4835 ± 0.2041^b^
Gavage group B5	5.7730 ± 0.5049^a^	59.9209 ± 2.2177^a^	2.9958 ± 0.2057^c^	14.1397 ± 1.0569^c^

*Different letters means significant difference (P <0.05); the same letter means the difference is not significant (P > 0.05)*.

#### Delayed Allergic Reaction in Mice

On day 15 of gavage administration, the differences between the left and right ears of mice in groups A3, A4, and A5 were significantly different from that in group A1 (*P* < 0.05), the difference between groups A2, A3, and A4 was also significant (*P* < 0.05). That in group A3 was 19.05% higher than that in group A2. On day 30 of continuous gavage administration, there was no significant difference in the left–right ear difference between any two groups, though that of group B5 was 10.22% higher than that of group B1 (Table 2).

**Table 2:** Effects of *R. mociliginosa* on delayed hypersensitivity (DTH) in mice.

**Groups**	**Concentrations of *Rhodotorula mucilaginosa* suspensions (CFU/ml)**	**The 15th the differences between the left and right ears of mice (g) (A group)**	**The 30th the differences between the left and right ears of mice (g) (B group)**
Control group A1, B1	0	0.0147 ± 0.0009^a^	0.0137 ± 0.0009^a^
Gavage group A2, B2	2 × 10^6^	0.0145 ± 0.0001^a^	0.0125 ± 0.0004^a^
Gavage group A3, B3	2 × 10^7^	0.0175 ± 0.0005^c^	0.0139 ± 0.0018^a^
Gavage group A4, B4	2 × 10^8^	0.0124 ± 0.0004^b^	0.0148 ± 0.0003^a^
Gavage group A5, B5	2 × 10^9^	0.0133 ± 0.0008^b^	0.0151 ± 0.0001^a^

*Different letters means significant difference (P <0.05); the same letter means the difference is not significant (P > 0.05)*.

#### The Determination of the Clearance and Phagocytic Index

On day 15, the clearance index and phagocytic index of mice in the gavage groups were significantly higher than those in group A1 (*P* < 0.05), and the differences between all gavage groups were also significant (*P* < 0.05). In this study, the clearance index and phagocytic index of mice in the gavage groups were significantly higher than those in group A1. The phagocytic index of mice significantly increased as the concentration of *R. mucilaginosa* suspension given by gavage increased. The carbon clearance capacity of mice decreased with time but was still significantly higher than that in group A1, which indicated that *R. mucilaginosa* significantly improved the phagocytosis of macrophages in mice. These findings were consistent with the results of Dong et al., where the inactivated *Saccharomyces cerevisiae* and inactivated *Torulaspora delbrueckii* improved the phagocytosis of monocytes/macrophages in mice (Table 3).

**Table 3:** Effects of *R. mucilaginosa* on carbon clearance and phagocytic index in mice (15 days and 30 days).

**Groups**	**Concentrations of *Rhodotorula mucilaginosa* suspensions (CFU/ml)**	**Clearance index (k)**	**Phagocytic index (α)**
Control group A1	0	0.0341 ± 0.0008^a^	3.3661 ± 0.0503^a^
Gavage group A2	2 × 10^6^	0.0467 ± 0.0006^b^	6.1389 ± 0.0145^b^
Gavage group A3	2 × 10^7^	0.0592 ± 0.0006^c^	6.3395 ± 0.1012^c^
Gavage group A4	2 × 10^8^	0.0625 ± 0.0002^d^	8.2606 ± 0.1630^d^
Gavage group A5	2 × 10^9^	0.0830 ± 0.0007^e^	9.2032 ± 0.0772^e^
Control group B1	0	0.0239 ± 0.0010^a^	2.8826 ± 0.0093^a^
Gavage group B2	2 × 10^6^	0.0341 ± 0.0013^b^	6.3498 ± 0.0061^b^
Gavage group B3	2 × 10^7^	0.0458 ± 0.0017^b^	6.8795 ± 0.0106^c^
Gavage group B4	2 × 10^8^	0.0545 ± 0.0021^b^	7.0266 ± 0.0374^d^
Gavage group B5	2 × 10^9^	0.0631 ± 0.0011^b^	7.5920 ± 0.0300^e^

*Different letters means significant difference (P <0.05); the same letter means the difference is not significant (P > 0.05)*.

#### The Determination of IgG, IgA in Serum

On days 15 and 30, serum IgA and IgG were significantly different between groups A4, A5, B4, B5, and group A1 and B1 separately (*P* < 0.05). On day 15, the serum IgA level in mice increased with increasing concentration of *R. mucilaginosa* suspension, with 64.93% higher serum IgA in group A5 than in group A1 and 65.64% higher serum IgG in group A5 than in group A1, which suggested that gavage administration of *R. mucilaginosa* suspension quickly and significantly improved the levels of immunoglobulins in mice (Table 4).

**Table 4:** Effects of *R. mucilaginosa* on IgA and IgG content in mouse serum.

**Groups**	**Concentrations of *Rhodotorula mucilaginosa* suspensions (CFU/ml)**	**The 15th IgA level (mg/mL)(A group)**	**The 30th IgA level(mg/mL)(B group)**	**The 15th IgG level(mg/mL)(A group)**	**The 30th IgG level(mg/mL)(B group)**
Control group A1, B1	0	2.5673 ± 0.0916^a^	3.3627 ± 0.0469^a^	10.3027 ± 1.9912^ab^	11.3018 ± 1.0962^ab^
Gavage group A2, B2	2 × 10^6^	2.6603 ± 0.0855^a^	3.7419 ± 0.1600^a^	10.1633 ± 0.8767^a^	12.7712 ± 0.7564^a^
Gavage group A3, B3	2 × 10^7^	2.7666 ± 0.1260^a^	2.6077 ± 0.1501^a^	12.1197 ± 0.7642^b^	10.5247 ± 1.6056^b^
Gavage group A4, B4	2 × 10^8^	3.6380 ± 0.1674^b^	2.6163 ± 0.0853^b^	16.6862 ± 0.2963^c^	10.9951 ± 1.1513^c^
Gavage group A5, B5	2 × 10^9^	4.2343 ± 0.1099^c^	2.4504 ± 0.0458^c^	17.0653 ± 0.5931^d^	10.6381 ± 0.6151^d^

*Different letters means significant difference (P <0.05); the same letter means the difference is not significant (P > 0.05)*.

#### The Determination of IL-2, TNF-α, and INF-γ in Serum

On day 15, the serum IL-2 level in each gavage group was significantly higher than that in control group (*P* < 0.05), and that in group A5 was significantly higher than those in groups A2, A3, and A4 (*P* < 0.05). Serum IL-2 was highest in group A2, in which it was 37.68% higher than in group A1. On day 30, serum IL-2 in all gavage groups was higher than that in control group (*P* < 0.05), and that in group B2 and B3 was significantly different from those in groups B1, B4, and B5 (*P* < 0.05) (Table 5a). On day 15, serum TNF-α in all gavage groups was higher than that in group B1, and that in group A5 was significantly higher (by 27.06%) than that in group A1 (*P* < 0.05). On day 30, serum TNF-α in group A2 was significantly higher than that in all other groups (*P* < 0.05), being 22.69% higher in group B2 than in group B1 (Table 5b). On day 15, serum INF-γ in group A4 and A5 was significantly higher than that in groups A1, A2 and A3 (*P* < 0.05), with 42.77% higher in group A5 (highest) than in group A1. On day 30, serum INF-γ in groups B2, B4, and B5 was significantly higher than that in group B1 (*P* < 0.05), being 20.98% higher in group B2 (highest) than in group B1 (Table 5c).

**Table 5a:** Effects of *R. mucilaginosa* on IL-2 content in mouse serum.

**Groups**	**Concentrations of *Rhodotorula mucilaginosa* suspensions(CFU/ml)**	**The 15th IL-2 level (ng/mL)(A group)**	**The 30th IL-2 level (ng/mL)(B group)**
Control group A1, B1	0	42.2282 ± 1.0537^a^	45.4619 ± 4.9394^a^
Gavage group A2, B2	2 × 10^6^	58.1398 ± 5.6113^b^	54.9261 ± 3.3352^b^
Gavage group A3, B3	2 × 10^7^	57.2005 ± 1.6055^b^	56.3831 ± 2.6046^b^
Gavage group A4, B4	2 × 10^8^	56.8263 ± 4.6627^b^	47.1474 ± 2.1157^ac^
Gavage group A5, B5	2 × 10^9^	52.5900 ± 5.9948^c^	50.1478 ± 2.5127^ac^

*Different letters means significant difference (P <0.05); the same letter means the difference is not significant (P > 0.05)*.

**Table 5b:** Effects of *R. mucilaginosa* on TNF-α content in mouse serum.

**Groups**	**Concentrations of *Rhodotorula mucilaginosa* suspensions(CFU/ml)**	**The 15th TNF-α level (ng/mL)(A group)**	**The 30th TNF-α level (ng/mL)(B group)**
Control group A1, B1	0	121.1118 ± 5.7134^a^	118.8222 ± 12.6441^a^
Gavage group A2, B2	2 × 10^6^	122.0142 ± 10.5412^a^	145.7779 ± 11.1390^b^
Gavage group A3, B3	2 × 10^7^	123.5846 ± 9.9194^a^	119.2891 ± 14.5655^a^
Gavage group A4, B4	2 × 10^8^	136.5643 ± 10.5082^ab^	114.7225 ± 6.5545^a^
Gavage group A5, B5	2 × 10^6^	122.0142 ± 10.5412^a^	145.7779 ± 11.1390^b^

*Different letters means significant difference (P <0.05); the same letter means the difference is not significant (P > 0.05)*.

**Table 5c:** Effects of *R. mucilaginosa* on INF-γ content in mouse serum.

**Groups**	**Concentrations of *Rhodotorula mucilaginosa* suspensions (CFU/ml)**	**The 15th IFN-γ level (ng/mL)(A group)**	**The 30th IFN-γlevel (ng/mL)(B group)**
Control group A1, B1	0	120.1411 ± 5.7681^a^	136.4278 ± 12.4568^a^
Gavage group A2, B2	2 × 10^6^	125.0461 ± 7.0326^a^	165.0500 ± 6.3586^b^
Gavage group A3, B3	2 × 10^7^	124.1260 ± 8.0205^a^	126.7498 ± 7.6461^ac^
Gavage group A4, B4	2 × 10^8^	156.4690 ± 5.2370^b^	116.8725 ± 7.5671^c^
Gavage group A5, B5	2 × 10^9^	171.5170 ± 6.1554^c^	117.7812 ± 3.6161^c^

*Different letters means significant difference (P <0.05); the same letter means the difference is not significant (P > 0.05)*.

#### The Determination of CD4 and CD8 in Serum

On day 15, serum CD4 in groups A4 and A5 was significantly higher than that in group A1, A2, or A3 (*P* < 0.05), being 28.48% higher in group A5 (highest) than in group A1. On day 30, the serum CD4 levels in all gavage groups were significantly different from that in group B1 (*P* < 0.05), being 14.00% higher in group B2 (highest) than in group B1. On day 15, the serum CD8 levels in all gavage groups were higher than that in group A1, being 38.42% higher in group A5 (highest) than in group A1. Although there was a slight difference in the change in CD8 levels in gavage groups on day 30, CD8 was significantly different between groups B5 and B1 (*P* < 0.05). On day 15, the CD4/CD8 ratios in groups A3, A4, and A5 were higher than that in group A1, being 61.83% higher in group A5 (highest) than group A1. On day 30, the CD4/CD8 ratios in all gavage groups were higher than that in group B1, being 47.62% higher in group B5 (highest) than in group B1 (Table 6).

**Table 6:** Effects of *R. mucilaginosa* on CD4 and CD8 content in mouse serum.

**Groups**	**Concentrations of *Rhodotorula mucilaginosa* suspensions (CFU/ml)**	**The 15th CD4 level (ng/mL) (A group)**	**The 30th CD4 level (ng/mL) (B group)**	**The 15th CD8 level (ng/mL)(A group)**	**The 30th CD8 level (ng/ mL) (B group)**
Control group A1, B1	0	204.8211 ± 2.7772^a^	223.8303 ± 7.4948^a^	205.7741 ± 3.5213^a^	198.0364 ± 5.8024^a^
Gavage group A2, B2	2 × 10^6^	205.6987 ± 3.7672^a^	255.1647 ± 4.4869^b^	207.9286 ± 3.6817^a^	205.1262 ± 3.7478^a^
Gavage group A3, B3	2 × 10^7^	206.843 ± 1.9465^a^	199.1808 ± 3.1151^c^	206.4245 ± 5.9868^a^	184.6212 ± 6.6930^b^
Gavage group A4, B4	2 × 10^8^	231.1466 ± 1.9475^b^	191.2466 ± 9.2754^c^	252.4780 ± 10.8083^b^	196.1165 ± 3.8671^a^
Gavage group A5, B5	2 × 10^9^	263.1465 ± 6.2777^c^	177.6959 ± 3.0881^d^	284.8292 ± 8.1849^c^	185.6893 ± 3.5535^b^

*Different letters means significant difference (P <0.05); the same letter means the difference is not significant (P > 0.05)*.

### Effect of *Rhodotorula mucilaginosa* on the Gut Microbiota of Mice

Samples from mice in the control group (A1) and gavage groups (A2, A3, A4, and A5) on day 15 and samples from mice in the control group (B1) and gavage groups (B2, B3, B4, and B5) on day 30 were collected to obtain 809,991 high-quality gene sequences. A total of 6,172 OTUs were obtained by clustering, with 97% similarity (Table 7a). A total of 394,590 high-quality gene sequences were obtained in group A, with 3,783 OTUs; 415,401 high-quality gene sequences were obtained in group B, with 2,389 OTUs. Between group A1 and A2, group A1 had the highest number of high-quality gene sequences and highest level of clustering, which were 85,047 high-quality gene sequences and 837 OTUs, respectively. Between group B1 and B2, group B1 had the highest number of high-quality gene sequences (86,936), while group B2 had the highest number of OTUs (692), which was significantly lower that of group A. Subsequent analysis was carried out after annotation of representative sequences of OTUs, and the library coverage was 99.56–99.75%.

**Table 7a:** Date statistics of sample gene sequences.

**Groups**	**E1**	**A1**	**A2**	**A3**	**A4**	**E2**	**B1**	**B2**	**B3**	**B4**	**Total**
Sequences	85,047	78,645	73,719	76,485	80,694	79,310	83,190	81,071	86,936	84,893	809,991
OTUs	837	717	710	758	761	588	692	642	405	620	6,172
The relative abundance (%)	99.70	99.60	99.56	99.67	99.64	99.71	99.68	99.69	99.75	99.73	–

#### The Dilution Curve of OTU Number

**Figure 2:**
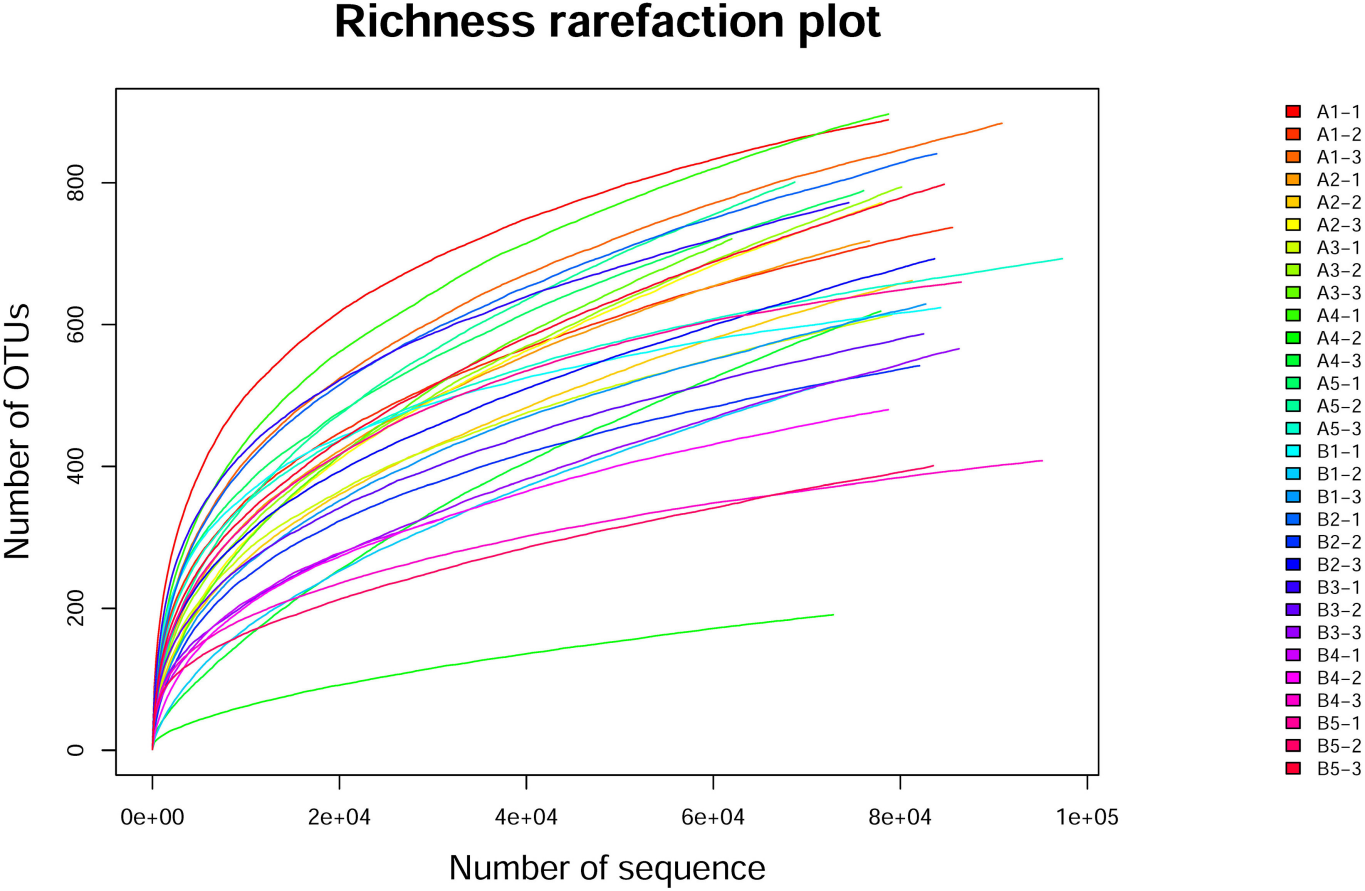
Alpha exponential dilution curve. The dilution curve was drawn with the number of sequences sequenced (as generated by random sampling) as the abscissa and its corresponding OTU number based on 97% sequence similarity as the ordinate. The dilution curve was infinitely close to the maximum number of OTUs contained in the sample, but species abundance differed between samples. All curves flattened and approached a plateau.

To evaluate the quality of sequencing results of the samples and the reliability of data, the dilution curve was drawn with the number of sequences sequenced (as generated by random sampling) as the abscissa and its corresponding OTU number based on 97% sequence similarity as the ordinate (Figure 2). The detail data was showed in Supplement 1. The dilution curve was infinitely close to the maximum number of OTUs contained in the sample, but species abundance differed between samples. The upward trend of all curves flattened and approached a plateau, indicating that the data volume for sequencing was reasonable and reliable, and a larger data volume had little impact on the number of OTUs identified.

#### Analysis of Intestinal Microflora Diversity in Mice (Alpha Diversity)

**Figure 3:**
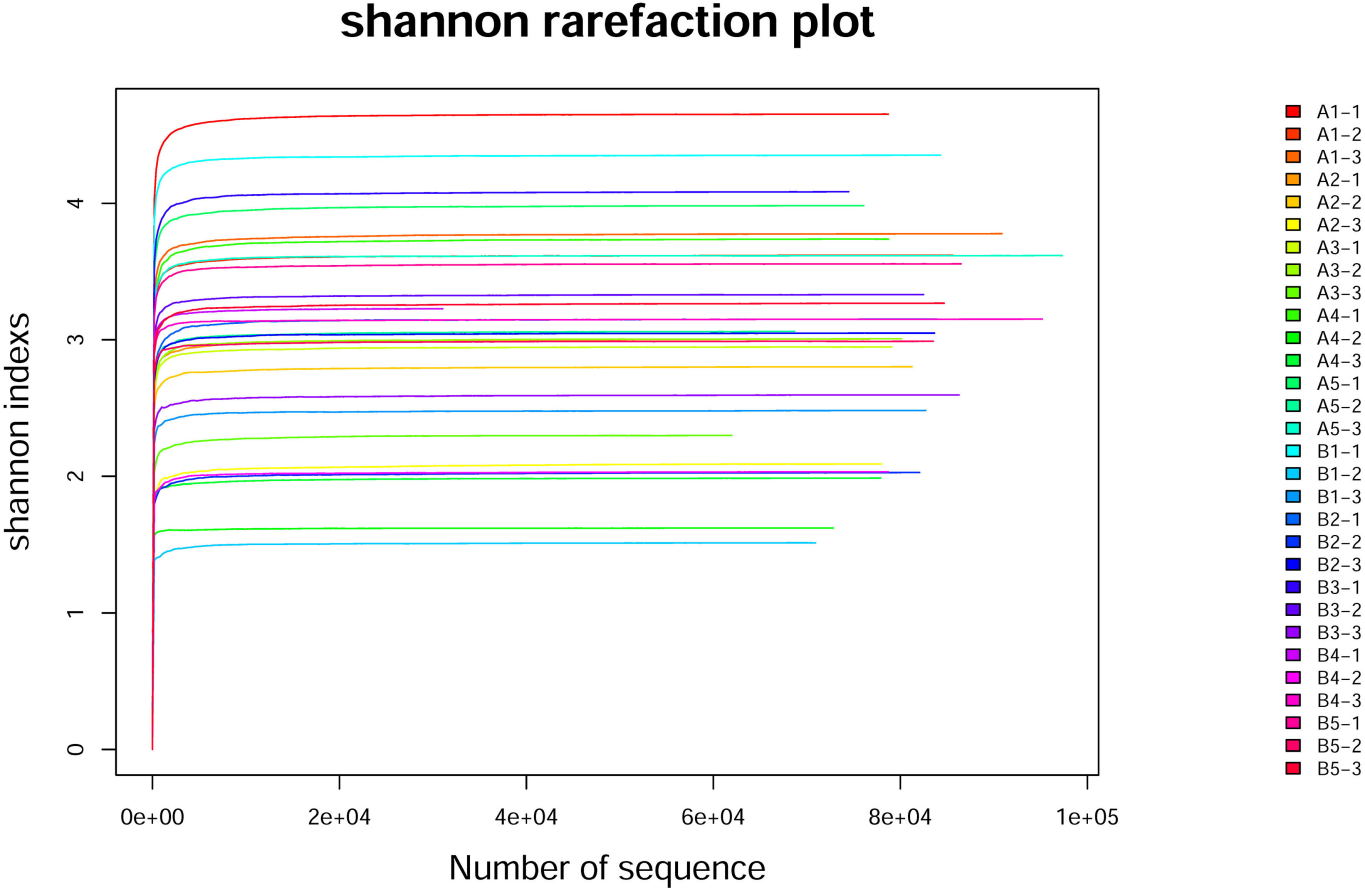
Shannon rarefaction plot curve. The horizontal axis is the number of randomly selected sequences in the sample, the vertical axis is the corresponding Alpha index obtained, and each curve is a sample. A1-1, A1-2, A1-3, A1-4, and A1-5 indicate that group A1 was sequenced three times; B1-1, B1-2, B1-3, B1-4, and B1-5 indicate that group B1 was sequenced three times.

The dilution curves of the Shannon index of sequenced samples were drawn for analysis, as shown in Figure 3 (**Table 8**). The detail data was showed in Supplement 2. These curves all rapidly rose at first and then tended to flatten out to a steady state, which indicated that data volume chosen for sample sequencing was sufficient for the subsequent analysis. The values of the alpha diversity index of gut microbiota in samples of the intestinal contents of mice are shown in Table 7b. The values of coverage in the control group and gavage groups were above 99.5%, indicating that data volume for sequencing in the study was sufficient and that the sequencing results could represent real-world samples.

**Table 7b:** Diversity analysis of intestinal microflora in mice.

**Groups**	**Shannon index**	**Simpson index**	**ACE index**	**Chao1 index**	**Coverage**
E1	2.56	0.197	1,300	1,173	0.9970
A1	2.62	0.191	1,675	1,257	0.9960
A2	2.75	0.164	1,748	1,275	0.9956
A3	2.85	0.218	1,364	1,161	0.9967
A4	3.55	0.077	1,432	1,171	0.9964
E2	2.78	0.186	1,255	991	0.9971
B1	2.84	0.107	1,481	1,212	0.9968
B2	3.03	0.120	1,553	1,154	0.9969
B3	2.99	0.139	1,656	1,167	0.9975
B4	3.27	0.098	1,496	1,118	0.9973

Shannon index in all gavage groups was higher than that in the control group and increased with the concentration of *R. mucilaginosa* in the suspension administered by gavage (except in group B3). The highest Shannon index values were in A4 and B4, which were 38.67 and 17.63% higher than that in the control group, respectively. The Simpson index in the gavage groups (except group A3) was lower than that in the control group. The lowest Simpson index values were in groups A4 and B4, which were 60.71 and 47.31% lower than that in the control group, respectively. The Shannon index was higher and the Simpson index was lower for the gut microbiota of the gavage groups, the most significant difference coming in the group receiving the suspension with the highest *R. mucilaginosa* concentration. These findings indicate that *R. mucilaginosa* increased the diversity of the gut microbiota of mice.

On days 15 and 30 of gavage administration, the ACE index of gut microbiota of the gavage groups was higher than that in the control group, the highest being in groups A2 and B3, which were 34.46 and 31.95% higher than that in the control group, respectively. On day 15 of gavage administration, the Chao1 index of the gut microbiota was higher in two gavage groups and lower in the other two gavage groups than in the control group. On day 30 of gavage administration, the Chao1 index of gut microbiota in each gavage group was higher than that in the control group, the greatest difference being 22.30% for group B1. Both the ACE index and the Chao1 index were higher in gavage groups, indicating that the evenness and abundance of gut microbiota of mice in the gavage groups were significantly greater than they were in the control.

#### Phylum Level Analysis of Microbial Community

**Figure 4:**
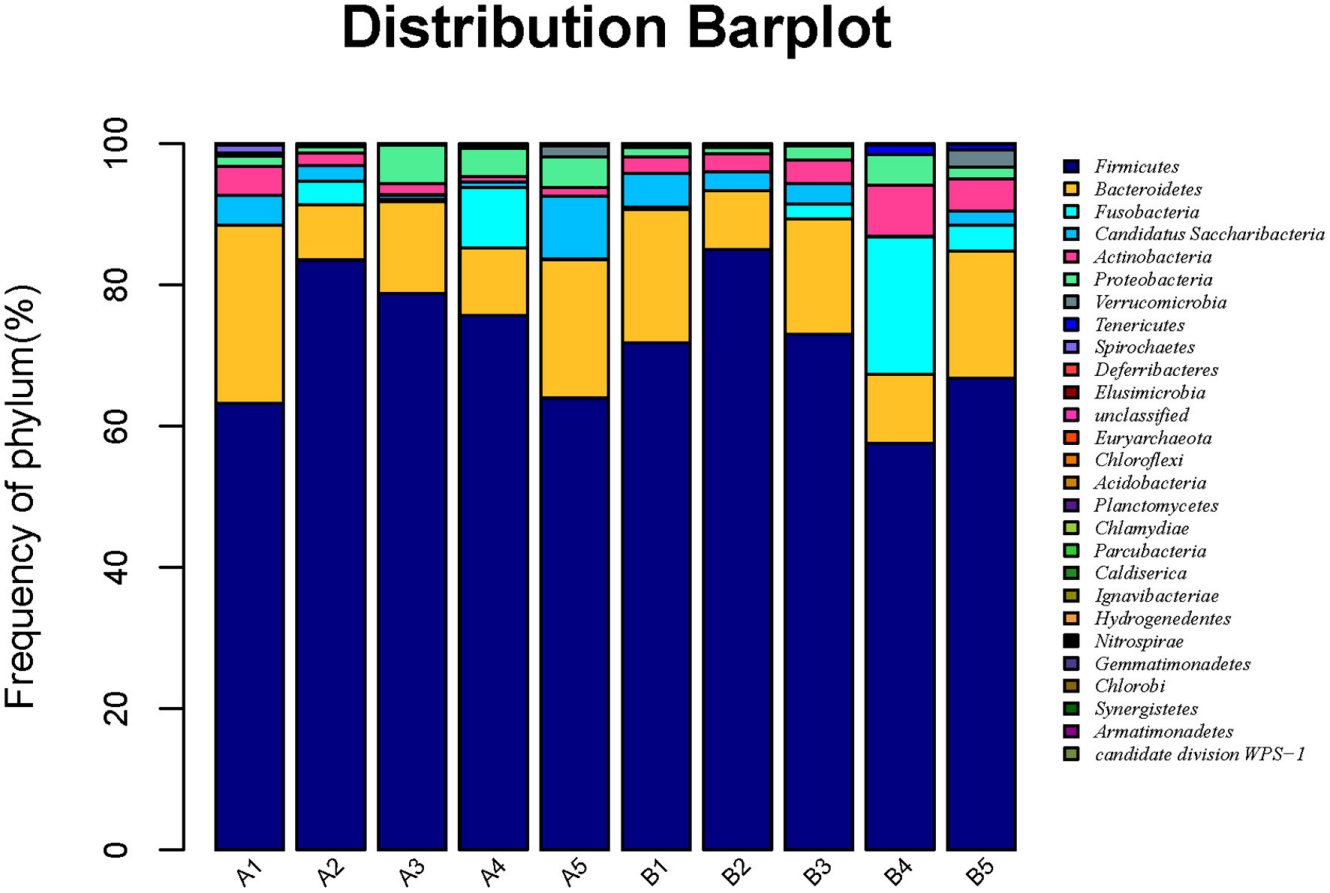
Horizontal abundance of intestinal microflora communities in mice at the phylum level. Clustering was conducted according to the abundance of OTUs, and different colors were used to represent high- and low-abundance microbial species to annotate OTUs to species.

Based on the results of OTUs, clustering was conducted according to the abundance of OTUs, and different colors were used to represent high- and low-abundance microbial species to annotate OTUs to species. The microbiota in intestinal contents of mice in 10 samples was statistically analyzed at the phylum level, as shown in Figure 4. The detail data was showed in Supplement 3 and Table 7c. The top six dominant phyla were *Firmicutes, Bacteroidetes, Fusobacteria, Candidatus Saccharibacteria, Actinobacteria*, and *Proteobacteria*. The dominant phylum in groups A and B was *Firmicutes*, and the secondary phylum (except in group B4) was Bacteroidetes. The gavage groups had more diverse phyla than the control group at different times, *Firmicutes, Bacteroidetes, Candidatus Saccharibacteria*, and *Actinobacteria* being the dominant phyla in the gavage groups. *R. mucilaginosa* seemed to change the structure of the gut microbiota of mice at the phylum level but did not change the dominant phyla. In group A, the relative abundance of *Firmicutes* in groups A2 (83.8%), A3 (78.9%), A4 (75.8%), and A5 (63.9%) was higher than that in group A1 (63.3%); the relative abundance of *Bacteroidetes* in groups A2 (4.0%), A3 (11.1%), A4 (4.8%), and A5 (8.7%) was lower than that in group A1 (15.1%). In group B, the relative abundance of *Firmicutes* in groups B3 (85.0%) and B4 (73.0%) was higher than that in group B2 (71.9%), and the relative abundance in the other two gavage groups were lower than that in the control group. However, the relative abundance of *Bacteroidetes* in the four gavage groups was also lower than that in the control group. These data suggest that *R. mucilaginosa* increased the abundance of *Firmicutes* and decrease the abundance of *Bacteroidetes* in the intestinal contents of mice but did not change the dominant phyla (Table 8).

**Table 7c:** Relative abundance of species at the phylum level of intestinal microflora structure in mice (%).

**Phylum**	**E1**	**A1**	**A2**	**A3**	**A4**	**E2**	**B1**	**B2**	**B3**	**B4**
*Firmicutes*	63.3	83.8	78.9	75.8	63.9	71.9	85.0	73.0	57.4	66.9
*Bacteroidetes*	15.1	4.0	11.1	4.8	8.7	8.8	4.1	7.2	4.8	8.1
*Fusobacteria*	^#^	3.4	^#^	8.8	^#^	^#^	^#^	2.2	19.7	4.0
*Candidatus Saccharibacteria*	4.4	2.1	0.6	0.8	9.1	5.2	2.8	2.9	^#^	2.1
*Actinobacteria*	4.2	2.0	2.2	0.7	1.1	2.3	2.5	3.4	7.3	4.2
*Proteobacteria*	1.5	0.9	5.0	3.9	4.4	1.3	0.8	1.0	4.4	1.7

# *Suggested the Relative abundance of species below 0.1%*.

**Table 8:** Diversity analysis of intestinal microflora in mice.

**Group**	**Shannon index**	**Simpson index**	**ACE index**	**Chao1 index**	**Coverage**
A1	2.56	0.197	1,300	1,173	0.9970
A2	2.62	0.191	1,675	1,257	0.9960
A3	2.75	0.164	1,748	1,275	0.9956
A4	2.85	0.218	1,364	1,161	0.9967
A5	3.55	0.077	1,432	1,171	0.9964
B1	2.78	0.186	1,255	991	0.9971
B2	2.84	0.107	1,481	1,212	0.9968
B3	3.03	0.120	1,553	1,154	0.9969
B4	2.99	0.139	1,656	1,167	0.9975
B5	3.27	0.098	1,496	1,118	0.9973

#### Genus Level Analysis of Microbial Community

**Figure 5:**
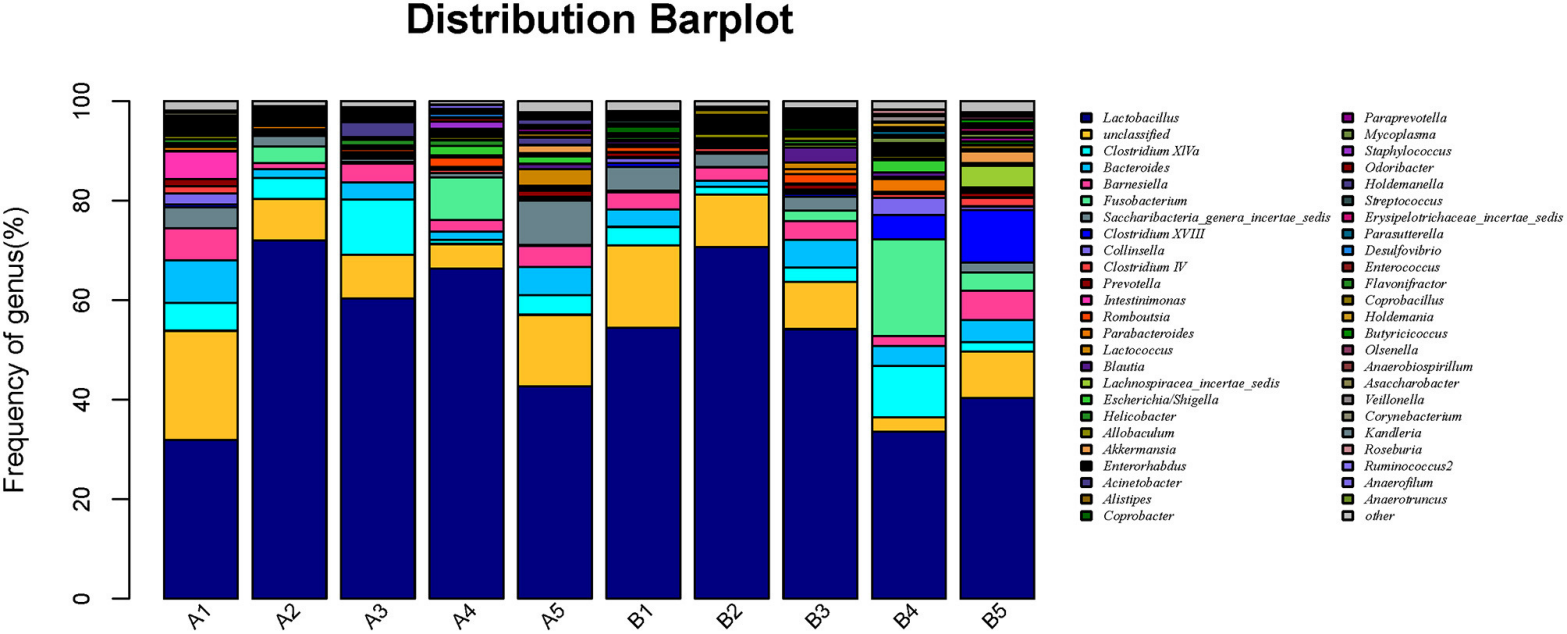
Horizontal abundance of intestinal microflora communities in mice at the genus level. The dominant genera were list from top to low. The horizontal axis is the number of each sample, and the vertical axis is the relative abundance ratio. Colors correspond to species names at this taxonomic level. The different colors and block width represents the relative abundance ratio of different species.

Microbiota in 10 samples were statistically analyzed at the genus level, as shown in Figure 5. The detail data was showed in Supplement 4. The top 7 dominant genera were *Lactobacillus, Clostridium, Bacteroides, Fusobacterium, Barnesiella*, and *Saccharobacter*; the most dominant genus in groups A and B was *Lactobacillus*, and the relative abundance of *Lactobacillus* in the gut microbiota of mice in all gavage groups (on day 15) was higher than that in the control group.

**Figure 6:**
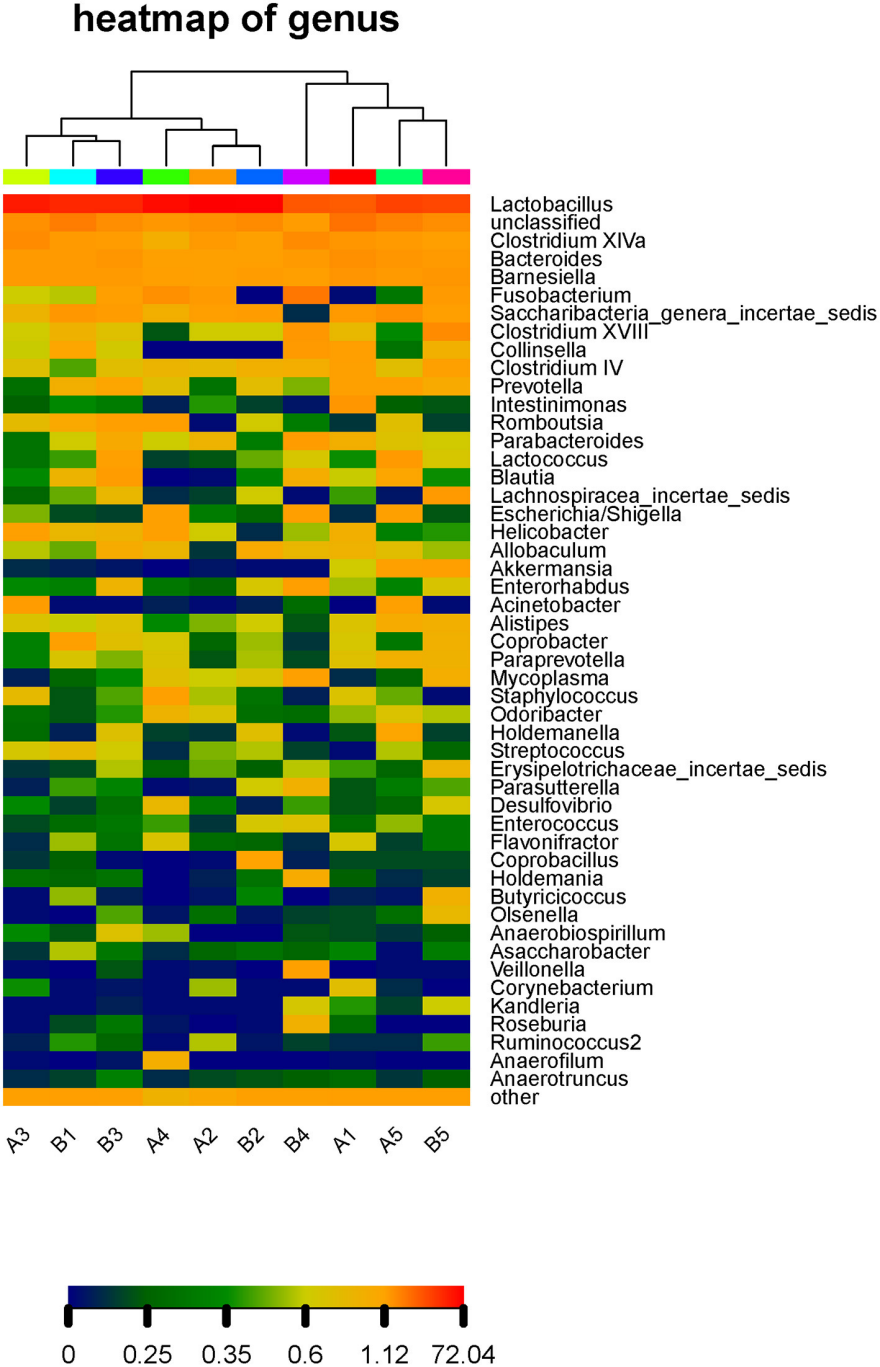
Thermal map of species abundance at the genus level in the intestinal microflora of mice. The abundance distribution of the taxa of the microbiota at different taxonomic levels, a heat map of species abundance was built for the microbiota with the top 50 relative abundance values in the gut microbiota of our mice and the rest of the species are grouped into Other. The horizontal axis is the number of each sample, and the vertical axis is the relative abundance ratio. Each column represents a sample, rows represent community structure, and color blocks Represents the relative species abundance value, the red color means the higher the relative abundance, the blue color and vice versa. In addition, the heat map is aggregated on the sample, the more similar the distribution of sample flora is, the closer the sample distance is, and the closer the position in the cluster tree at the top of the figure is., which is shown at the top of the graph. The samples from the same group are the same color.

To clearly reflect the abundance distribution of the taxa of the microbiota and to further reflect the diversity of the microbiota distribution at different taxonomic levels, a heat map of species abundance was built for the microbiota with the top 50 relative abundance values in the gut microbiota of our mice (Figure 6). *Lactobacillus, Clostridium, Bacteroides*, and *Barnesiella* had high relative abundance in all mice. There was little difference in the gut microbiota between the control group and gavage groups.

The gut microbiota structure of mice was altered at the phylum and genus levels by *R. mucilaginosa* ZTHY2, which led to an increased relative abundance of *Firmicutes* and *Lactobacillus* and a decreased relative abundance of *Bacteroidetes*. That is, *R. mucilaginosa* ZTHY2 increased the beneficial intestinal bacteria and reduced the harmful intestinal bacteria in mice.

## Discussion

The research on and application of *Rhodotorula* as microecologics have mainly focused on aquatic animals, and there are few relevant reports on livestock or poultry. *Rhodotorula*, especially *Rhodotorula benthica*, is a probiotic that has a wide range of sources, is easy to isolate and screen, is easy to culture, grows and reproduces fast, and is rich in metabolic products. It has nutritive functions, immunity-promoting effects, antioxidant functions, and high tinting strength for animals. Production of microecologics with *Rhodotorula* can have a broad market and bring great economic benefits (Ueno et al., 2011).

In this study, the culture medium, culture temperature, culture time, culture medium pH value, inoculum density, shaking speed during cultivation, and liquid volume medium were optimized by single-factor experiments, and the optimal culture conditions obtained from single-factor experiments were validated (Chen et al., 2017). We concluded that the best conditions for improving the production of carotenoids by *R. mucilaginosa* were as follows: culture temperature of 30°C, culture time of 60 h, medium pH value of 6, inoculum density of 6%, shaking speed of 180 r/min during cultivation, and medium fluid volume 60 mL/250 mL. Under these conditions, production of carotenoids reached 1.3658 g/L, and biomass reached 8.6530 g/L, which are 85.8 and 66.8% higher than those before optimization, respectively, providing evidence for *R. mucilaginosa* ZTHY2 as a carotenoid-producing strain.

The weight of immune organs is related to number of immune cells, so the immune organ index (immune organ weight/live weight) can be used to reflect the size of an immune organ, to reflect the immune function of the body to a certain extent (Chew and Park, 2004; Katsuura et al., 2009; Park et al., 2010). In this study, the spleen index and thymus index of mice higher than those of the control group and the difference was significant. Dong et al. found that inactivated Saccharomyces cerevisiae and inactivated *Torulaspora delbrueckii* had no significant effect on the spleen index or thymus index of mice. This discrepancy may be explained by the different yeast strains and treatment methods used in our two studies. Cytokines are a category of bioactive molecules produced by immune cells and other relevant cells (Yasui et al., 2011). In the process of an immune response, immune cells participate in and regulate the immune response by mutual stimulation and constraints with cytokines to keep the immune system in balance. IL-2 (Malek, 2003), TNF-α, INF-γ, CD4, and CD8 are important cytokines in the body. In the study by Dong et al., the mice were intravenously injected with inactivated Saccharomyces cerevisiae, and the results showed that serum TNF-α and IFN-γ were significantly increased in these mice (Dong, 2018). The findings are in line with ours, but the species and types of yeast used in the two studies are different. Therefore, further research is needed.

High-throughput sequencing, also known as next-generation sequencing, has been applied to the study of gut microbial diversity, making it possible to analyze the transcriptome and genome of a species meticulously and comprehensively; it is a new approach to study the microbial community structure in depth (Di Segni et al., 2018; Grosheva et al., 2020). In this study, after mice were intragastrically administered four concentrations of *R. mucilaginosa* suspensions, the microbiota in their intestinal contents was sequenced through 16S rDNA high-throughput sequencing to investigate the effects of *R. mucilaginosa* on the gut microbiota of mice. The upward trend of dilution curves of the OTU number and the Shannon index flattened, indicating that the data volume for sequencing was reasonable and reliable and could satisfy the requirements of subsequent effectiveness analysis.

In community ecology, alpha diversity analysis can reflect the diversity and richness of microbial communities. A high abundance and diversity of gut microbiota can effectively promote the absorption of nutrients by organisms and stimulate immune responses and disease prevention (Maynard et al., 2012; Hold and Hansen, 2019; Mirza et al., 2020). The diversity and abundance of microbial communities were analyzed with statistical indices, including the Chao1 index, ACE index, Shannon index, Simpson index, and OTU coverage. In this study, the coverage was as high as 99.5%, indicating a high probability of detecting the sequences of the gut microbiota of mice and the saturated sequencing depth (almost all species were detected). The increase in the Shannon index and the decrease in the Simpson index in the gavage groups were most prominent in mice receiving the highest concentration of *R. mucilaginosa* suspension, indicating that *R. mucilaginosa* could increase the diversity of the gut microbiota of mice (Chen et al., 2013).

In conclusion, this study had shown that carotenoid production can be significantly increased by optimizing the medium composition and culture conditions for *R. mucilaginosa* ZTHY2 and provided evidence to support the development and application of *R. mucilaginosa* ZTHY2 as a microecologics for animals and as a strain for carotenoid production. The strain *R. mucilaginosa* ZTHY2 boosts the immune function of mice and enriches and improves their gut microbiota structure, however, the mechanism of the *Rhodotorula mucilaginosa* as the probiotics in the gut need to be deeply research.

## Data Availability Statement

The original contributions presented in the study are included in the article/Supplementary Material, further inquiries can be directed to the corresponding author/s.

## Ethics Statement

The animal study was reviewed and approved by Laboratory Animal Centre of Guangdong Ocean University.

## Author Contributions

YG contributed on writing article. KH contributed on experiment. WX contributed on giving a hand in experiment. CX contributed on major guiding the experiment and support the fund. QY contributed on experiment and revised the manuscript. YL guiding the experiment. All authors contributed to the article and approved the submitted version.

## Conflict of Interest

The authors declare that the research was conducted in the absence of any commercial or financial relationships that could be construed as a potential conflict of interest.

## Publisher's Note

All claims expressed in this article are solely those of the authors and do not necessarily represent those of their affiliated organizations, or those of the publisher, the editors and the reviewers. Any product that may be evaluated in this article, or claim that may be made by its manufacturer, is not guaranteed or endorsed by the publisher.
